# Characteristics and duties of clinical research nurses: a scoping review

**DOI:** 10.3389/fmed.2024.1333230

**Published:** 2024-01-18

**Authors:** Ying Xing, Xinxin Wang, Chengrui Zhang, Weian Yuan, Xinlin Chen, Wei Luan

**Affiliations:** ^1^Nursing Department, Shuguang Hospital Affiliated to Shanghai University of TCM, Shanghai, China; ^2^Shanghai Jiao Tong University School of Nursing, Shanghai, China; ^3^GCP Center, Shuguang Hospital Affiliated to Shanghai University of TCM, Shanghai, China; ^4^Hospital Management Office of Shanghai University of TCM, Shanghai, China

**Keywords:** nursing role, clinical research nurses, research nurses, competency, clinical competence

## Abstract

**Introduction:**

The characteristics and duties of clinical research nurses (CRNs) are constantly developing and changing with the progress of medical technology and increasing needs in patient care. With the continuous deepening and standardization of clinical trials, the importance and status of CRNs during the whole process of clinical trials are also increasingly valued.

**Methods:**

A scoping review of studies related to the characteristics and duties of CRNs was conducted to clarify relevant roles and concepts. An electronic search was conducted on three English databases (PubMed, Web of Science, Embase) and two Chinese databases (CNKI and Wanfang database) in December 2023. Two authors independently screened the literature, extracted information from the included literature, and summarized and reported the findings.

**Results:**

A total of 26 articles published between 1991 and 2023 were analyzed, and four characteristics of CRNs were identified as participants and managers of clinical trials, caregivers and protectors of subjects, coordinators of research teams, and educators. Basic knowledge, skills and literacy, communication and coordination ability, and advanced research ability are the competencies required for CRNs.

**Conclusion:**

Further studies should focus on the importance of various characteristics of CRNs, so as to improve the quality of clinical trials and promote clinical evidence-based practice.

## 1 Introduction

As part of medical research and health research, clinical trial is a prospective evaluation of therapeutic interventions by volunteers or patients who are willing to accept drugs and different treatment techniques (1), with the aim of establishing basic theories on human disease mechanisms, disease prevention, and health promotion (2). Nurses are considered to be an essential presence in clinical trial teams, as they can support clinical activities and are responsible for the coordination and management of research activities (3). The participation of clinical research nurses (CRNs) is considered to positively improve the quality of clinical trial and be an indispensable element in clinical trials (4, 5).

However, the characteristics of CRNs is still unclear. The National Institutes of Health Clinical Center (NIHCC) defined the nursing role in clinical trial as twofold: to provide nursing services for clinical trials and to coordinate and execute the trials on behalf of the principal investigator (6). On the other hand, the UK Clinical Research Collaboration (UKCRC) described CRNs as nurses who were primarily engaged in research in a clinical setting (7). Some studies have supplemented that CRNs were registered nurses trained for research promotion and subjects’ protection (8). In addition, CRNs were responsible for data collection and recruitment of subjects, and maintaining the integrity of research protocols (4, 9).

As the characteristics of CRNs have not yet been clarified and unified, the conflict of role identification may cause CRNs to lack self-confidence and feel isolated (10), which is not conducive to the development of CRNs. For the above reasons, this study aims to summarize the existing conceptual differences by conducting a scoping review to systematically explore the characteristics of CRNs.

## 2 Materials and methods

### 2.1 Research design

A scoping review aims to examine the scope and nature of existing research on a particular topic or issue and determine the value of conducting a comprehensive systematic review. A scoping review methodology is based on the framework outlined by Arksey and O’Malley. Scoping reviews use a descriptive summary of the existing quantitative and qualitative studies to summarize the research findings and identify gaps in the existing research (11) in order to address the review questions. This review aims to provide a comprehensive summary of the characteristics and duties of CRNs in the existing literature without critically evaluating the included studies.

This review focused on the following questions: (1) What are the characters of CRNs in healthcare settings? (2) What are the competencies required for CRNs?

### 2.2 Search methods

Articles were identified by searching five databases: Web of Science (Core Collection), PubMed, Embase, and the Chinese databases CNKI^1^ and Wanfang database.^2^ The search strategy was developed by the three researchers in consultation with each other, and the following search terms were used: research nurse coordinator; research nurse; nurse researcher; nurse scientist; nursing scientist, clinical trial nurse, and clinical research nurse (see Supplementary material). The search was conducted from their inceptions to December 19,2023. Inclusion criteria were: (1) Subjects are CRNs; (2) Literature in both English and Chinese providing information about the role or responsibilities of CRNs; and (3) Quantitative and qualitative articles and conceptual analyses. The reason for including these sources is to ensure that the research captures all existing knowledge about the functions of the CRNs role. The exclusion criteria are: (1) reviews, books, letters to the editor, and abstracts of speeches.

### 2.3 Data extraction and analysis

The data was extracted from the included literature and identified into themes related to nurses’ role, and competency by using an inductive approach. We use Microsoft Excel to record extracted data including author, title, year of publication, country, nurses’ role, and competency. We then identified roles, competency, and fractionized them into themes related to procedures of clinical trial. Ongoing cross-checks were conducted by the first and third authors to ensure the accuracy of data extracted from a random sample of included papers, and disagreements were confirmed through mutual consensus by conducting an online meeting of 4 people (first author, second author, third author and one of the corresponding author) to discuss disagreements and review the article together if necessary to determine how to extract the data. We analyzed the results and presented them in narrative text and tabular form.

## 3 Results

### 3.1 Search outcome

**Figure 1:**
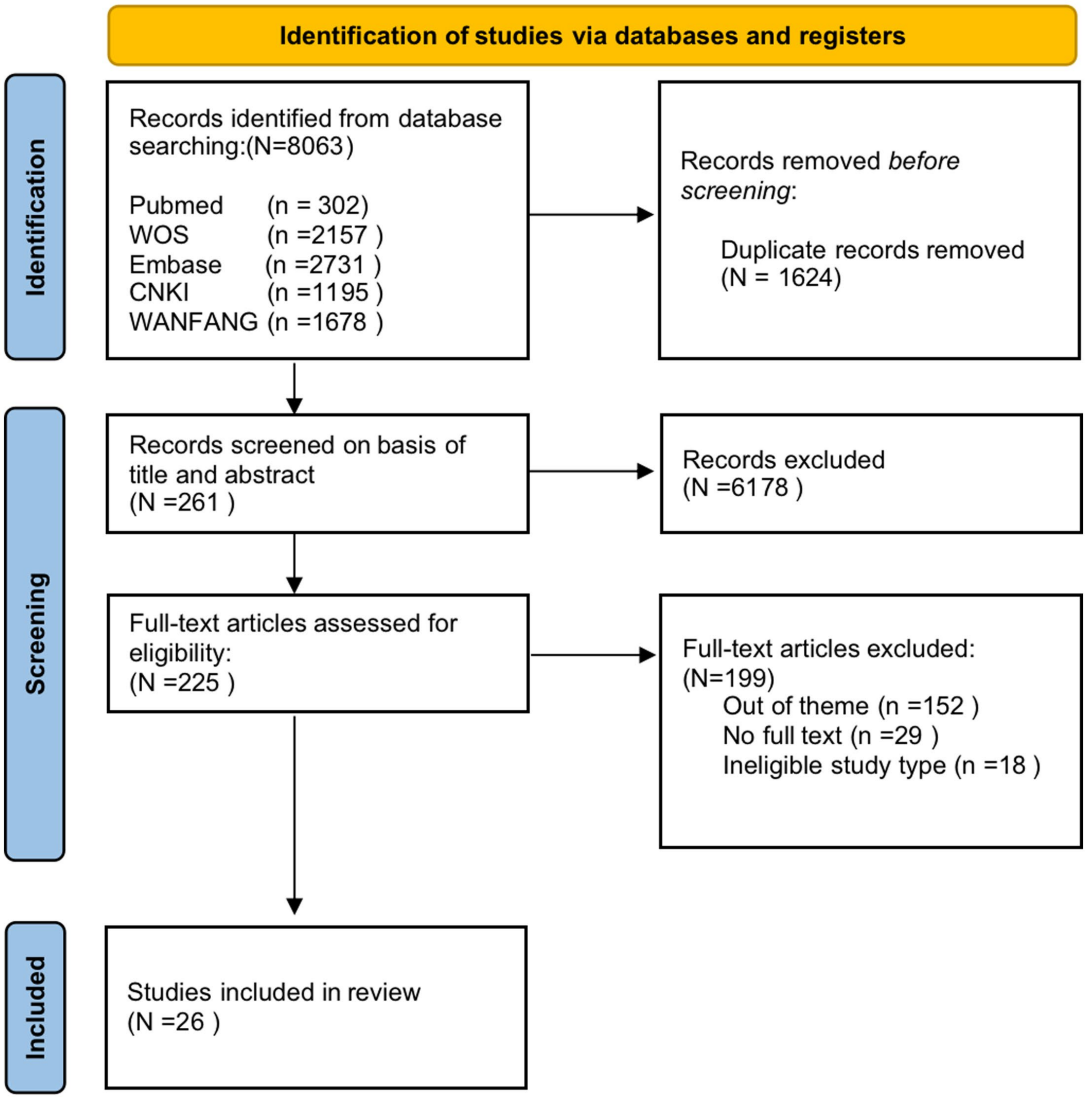
Flowchart of the literature screening process.

A total of 8,063 articles were obtained from the initial search of Chinese and English databases, as shown in Figure 1. The literature search results were reviewed, and duplicate results were excluded using Endnote 20, leaving 6,439 articles. According to the inclusion and exclusion criteria of the study, two authors independently scrutinized the titles and abstracts of the articles, leaving 261 articles remained to be screened in full text. If two reviewers had doubts, the full version was analyzed independently. A third researcher was asked to assist in the judgment if there was any disagreement. After screening the full-text articles, 26 articles were finally included. The dataset for this scoping review was constructed by extracting findings relevant to the research question.

### 3.2 General characteristics of the included literature

The 26 included articles were published between 1991 and 2023 (Table 1), most of which were published after 2010 (*n* = 22, 84.62%), and the role of CRNs began to receive more attention in the past 5 years. The articles came from seven countries (based on the affiliation of the first author), with the top three being the United States (*n* = 9, 34.62%) (4, 9, 12–18), the United Kingdom (*n* = 7, 29.17%) (19–25), and China (*n* = 4, 16.67%) (Liu et al., 2020; Li et al., 2020; Deng et al., 2023) (26). Among the 26 studies, research methods varied. Regarding research methods, 16 studies (66.67%) (Li et al., 2020), (4, 9, 14–23, 27–29) used qualitative methods and 10 studies (38.46%) (Liu et al., 2020; Deng et al., 2023) (12, 13, 24–26, 30–32) used quantitative methods. As for specific methods, survey interviews (*n* = 7, 29.17%), or focus groups (*n* = 6, 25%) were the main methods used.

**Table 1:** General characteristics of the included literature.

Authors, publication year, country	Type of study	Research theme	Main findings
Barthow et al. (2015), New Zealand	Qualitative study	Role of the research nurse	Explaining the purpose of the study, obtaining participant consent, using clinical assessment tools with research instruments, using information technology for data management and analysis.
Beer et al. (2022), Australia	Qualitative study	Role of the research nurse	Acting as an enabler to address systemic barriers; acting as a facilitator to address practitioner barriers; acting as a navigator to address subject barriers.
Bevans et al. (2011), United States	Quantitative research	Role of the research nurse	Providing direct clinical and research care to individual participants; clinical practice; facilitating communication with the research team, communicating the impact of study procedures on subjects, coordinating activities to minimize risk to participants.
Brant et al. (2015), United States	Qualitative research	Role of the research nurse	The mentor is the primary role; other roles include adjunct faculty at the local school of nursing, cancer committee members, and the principal investigator.
Carter et al. (2020), United States	Qualitative research	Role of the research nurse	Conducting and disseminating joint research, translating evidence into practice, developing formal education programs for health system nurses, scholarly activity between health systems, and increasing the visibility and valuation of postdoctoral nurses.
Cassidy et al. (1991), United States	Qualitative research	Role of the research nurse	Clinical trials, administrative tasks, adherence to protocol guidelines, conducting subject assessments, encouraging subject trust and reassurance, providing messaging, establishing ongoing open lines of communication, completing treatment protocols, toxicity observation and reporting, follow-up tracking, proper documentation of study records.
Catania et al. (2012), Italy	Quantitative study	Role of the research nurse	Much involvement in actual nursing care with little attention to data management and organizational activities. Involved in experimental drug administration, protocol implementation, partially involved in informed consent process.
Dube et al. (2017), United States	Qualitative research	Role of the research nurse	Assessment and planning of care related to clinical research before, during and after surgery.
Ecklund et al. (1999), United States	Qualitative research	Role of the research nurse	Protocol development and implementation, data management, subject tracking, communication, personnel management, organization and planning of staff meetings, fiscal management, and community outreach.
Gibbs et al. (2012), United Kingdom	Qualitative research	Role of the research nurse	Coordinating the day-to-day management of the trial and ensuring that the study is conducted in accordance with relevant legislation, research protocols and guidelines. The most recognized skills and responsibilities include screening, recruiting and obtaining informed consent from subjects and/or relatives.
Green et al. (2011), United Kingdom	Qualitative research	Role of the research nurse	Acting as a direct caregiver; being a key contact for trial participants; organizing research ethics committees and research and development submissions, and all related correspondences.
Hølge-Hazelton et al. (2016), United Kingdom	Qualitative research	Role of the research nurse	Use of knowledge in practice, clinical thinking and analytical skills, clinical judgment and decision-making skills, professional leadership and clinical investigation, coaching and mentoring skills, research skills and changing practice.
Johnson et al. (2010), United Kingdom	Qualitative research	Role of the research nurse	Effective communication with members of the multidisciplinary team and good relationships with all departments and trial participants involved in the trial.
Kao et al. (2015), China	Quantitative research	Role of the research nurse	The role related to “subject protection” had the highest level of agreement, followed by “study coordination and management,” “clinical care of subjects,” “clinical care of subjects,” “clinical care of subjects,” “clinical care of subjects,” and “clinical care of subjects,” and “advanced practice nursing functions.”
Lavender et al. (2019), United Kingdom	Qualitative research	Role of the research nurse	Clinical research nurses play an expert role in recruiting subjects and caring for subjects in clinical trials, which requires specialized education and training.
Ledger (2008), United Kingdom	Quantitative research	Competencies required of research nurses	Individual development framework: in collaboration with an external clinical research training consultant, a course specifically designed to equip research nurses with knowledge of regulatory requirements as well as trust processes and procedures, and guidance on drug development, trial design, intellectual property rights, and recruitment procedures.
Legor et al. (2021), United States	Qualitative research	Role of the research nurse	As advocates; as care coordinators; as educators.
Wilkes et al. (2012), Australia	Quantitative research	Role of the research nurse	Respondents’ role domains had the highest frequency scores for the informed consent process, followed by implementation and evaluation, and data management.
Liu et al. (2020), China	Quantitative research	Role of the research nurse	Clinical practice; clinical trial management protocol; subject management; pharmaceutical management; specimen management; equipment management; documentation management; coordination; subject protection; education.
Li et al. (2020) China	Qualitative research	Competencies required of research nurses	A system of core competency indicators for research nurses was established: basic knowledge, skills, and literacy; clinical practice; study management; coordinated management and ongoing management of studies; human subjects protection; scientific contribution.
Deng et al. (2023), China	Quantitative research	Competencies required of research nurses	A training system for research nurses was constructed, consisting of four level of indicators: “clinical trial-related professional knowledge,” “nursing theoretical knowledge,” “nursing professional skills,” “professionalism,” 14 secondary indicators, and 56 tertiary indicators.
Veal et al. (2017), United Kingdom	Quantitative research	Role of the research nurse	Collection and handling of blood samples; transportation of clinical samples; administration of new drugs or procedures; administration of investigational medicines; informed consent of participating subjects; prescribing of investigational medicines in the clinical trial setting.
McCabe et al. (2019), United States	Qualitative Research	Role of the research nurse	Comprehensive care of participants; training and education of subjects and staff; contribution to clinical science; unique combination of clinical and critical thinking skills; clinical practice.
Mackle et al. (2019), New Zealand	Qualitative research	Role of the research nurse	Managing research trials; obtaining subject consent; balancing subject needs with research needs; bridging gaps to achieve research.
Fisher et al. (2022), United States	Quantitative research	Role of the research nurse	Monitoring study participants for potential adverse events; collecting study endpoint data; providing nursing leadership within the interdisciplinary team; participation in clinical, unit, and/or protocol rounds.
Backman Lönn et al. (2022), Sweden	Quantitative research	Role of the research nurse	CRNs are involved in pre-study activities, study implementation and evaluation to various degrees. Concerned informed consent and managing the investigational products were rated as most important.

### 3.3 Characters of the CRNs

We summarized four characters of CRNs (Table 2).

**Table 2:** Characters of the CRNs.

Role	Description	Citation
Participants and administrators of clinical trials	1. Participation in clinical trials: Management of clinical and research execution activities	(13, 16, 27)
	2. Follow uniform clinical guidelines	(13, 17)
	3. Clinical trial management Data management Specimen management File management Drug management Facility management Subject management	(4, 12, 13, 17, 19, 20, 25, 27, 30, 31, 39)
Caregivers and protectors of subjects	1. Direct care for subjects	(4, 13, 14, 16, 18, 23)
	2. Subject protection	(9, 12, 16, 19, 26, 27, 29, 30, 32, 39)
Coordinator of research teams	Facilitates interdisciplinary team communication and collaboration	(12, 16, 28, 29, 39)
Educator	Guide training and education	(9, 31, 39)

#### 3.3.1 Participants and administrators of clinical trials

CRNs participate in the clinical trial process and follow uniform clinical guidelines, in addition to assisting in the management of clinical trials such as data, specimens and documentations.

Participating in clinical trials means managing clinical and research execution activities. CRNs participate in initiation meetings and keep all required documents in place before clinical trial start (30). CRNs can apply theoretical expertise to complete the collection of study specimens, data, etc., using clinical assessment tools and research tools during the procedure of clinical trial (13, 27).

Nurse scientist maintain program of research and guide clinical teams through the complexities of developing and conducting a research study (33), similar with the role of principal investigator. The principal investigator is responsible for conducting a clinical trial and complete trial following series regulations (34, 35), but most of them are physicians or dentists. As principal investigators, nurse scientists can maintain program of search during clinical trials and identify nurses to mentor for research (14). As a mentor, nurse scientist also provides education about research methods and research protocol development (33). There are uniform clinical guidelines for how trials should be performed. CRNs were found to ensure that research was conducted in accordance with relevant legislation, research protocols and guidelines. In addition, CRNs should understand the protocol and ensure that other researchers understand the research protocol (13, 17) and carry out the research under the guidelines.

In terms of clinical trial management, CRNs mainly manage research data, specimens, documents, drugs, equipment and facilities, and also take subject management into consideration. For trial data, CRNs are required to collect endpoint data of specific study (12) and report high-quality data in a timely manner (17), while using information technology for data management and analysis (4, 27). Subject data protection (13) and paper and electronic data entry (20) are also required. Specimen management is also within the scope of CRN’ s duties. In addition to completing the collection of biological specimens, CRNs are responsible for handling biological specimens such as centrifugation and storage as well, and appropriate sample transportation (Liu et al., 2020) (25). Transcription of data from raw files to case report forms, organization of clinical trial folders, and maintenance of study documents are included in document management duties (Liu et al., 2020) (19). In terms of drug management, research nurses need to be responsible for the management and prescribing of experimental drugs (25). Liu et al., (2020) elaborated on the content of drug management. CRNs are partly responsible for receiving, distributing and recording experimental drugs with clinical trials pharmacist, and the remaining experimental drugs and medication record cards of subjects should be recovered after the trial. After checking the remaining drugs and empty packaging with the inspector, CRNs complete the counting and recording, and carry out the recycling and destruction. In addition, the management of research equipment and facilities also falls within the scope of the CRN’s managerial responsibilities, including the security of file cabinets, computers, centrifuges, and other equipment. In terms of subject management, CRNs were involved in the recruitment, screening, and enrollment of subjects (13, 31), and worked with physicians to check whether the existing test results of the subjects met the inclusion criteria and to arrange for any additional screening tests (Liu et al., 2020).

#### 3.3.2 Caregivers and protectors of subjects: providing clinical and research care for subjects

CRNs are required to fulfill the plan of care for subjects and ensure the safety of subjects, which is the most important task to implement. As caregivers, subjects are provided with direct and comprehensive care (13, 16, 18), and CRNs play an expert role in subject caring (23). Combined with their professional knowledge and skills, CRNs participate in the assessment and treatment of subjects, such as evaluating and planning clinical trial-related care before and after surgical treatment, and being familiar with the dosage and administration route of investigational drugs (4, 14, 16). In addition, CRNs need to give trust and comfort, provide emotional support, establish continuous and open communication channels for subjects to balance their nursing needs (16, 18), and improve follow-up tracking and proper documentation of the study process. For subject advocate, CRNs take into account subjects’ rights and interests, providing subjects’ protection to reduce subjects’ risks. Moreover, CRNs should respect and maintain subjects’ cultural customs and provide support. Among the characteristics and duties of CRNs, subject protection is the most recognized and implemented in clinical practice (26), in which obtaining subjects’ informed consent is the primary task (27, 29). The frequency score for the item of informed consent was the highest in one quantitative study (32), and informed consent was also rated as most important by CRNs (30). CRNs also need to coordinate activities to reduce risks to subjects and monitor their physical and psychological changes (16). Monitoring study participants for potential adverse events is one of the most occuring activities daily (12), CRNs need to identify and report any adverse events to the principal investigator in a timely manner to assist with their management (Liu et al., 2020) (19). Due to the diverse recruitment channels for subjects from different cultures, CRNs should coordinate research activities to provide spiritual support and cultural respect for subjects. For subjects with speech communication disorders caused by different cultural backgrounds, CRNs need to dispatch interpreters to advocate nursing for subjects in multicultural backgrounds. For subjects of different nationalities, research (9) also advocates social and spiritual support for subjects.

#### 3.3.3 Coordinator of research teams

CRNs are responsible for effective communication and coordination with trial sponsors and research team members, acting as coordinators with the research team, and promoting team communication and cooperation by providing nursing leadership within the interdisciplinary team (12). The study (22) found that the success of a clinical trial largely depends on effective communication with members of the multidisciplinary team, as well as good relationships with all departments and trial participants involved in the trial, including members of the ethics committee, research and development staff, clinical staff, pharmacists, etc. CRNs became an important liaison for trial participants, organizing all communications with the ethics committee, obtaining institutional and sponsor approval (16), liaising with the sponsoring company to negotiate study funding, and assisting in communication to determine the time and location of the kick-off meeting, as well as participants and other outreach efforts. CRNs were also involved in communication within the research team, coordinating research visits by researchers and facilitating the research team’s attention to subjects. In addition, CRNs are required to coordinate barriers, the barriers to clinical trial within the team and those encountered by participants in clinical trial (28). For investigator visits, CRNs coordinated, received and monitored the visit, coordinating the research physician’s time with the subject’s visit requirements (Liu et al., 2020). Mackle et al. (29) found that promoting the research team’s attention to subjects could balance the needs of subjects and research needs, so CRNs should strengthen the research team’s attention to subjects according to the findings of existed literature.

#### 3.3.4 Educator

CRNs are responsible for training new team-members in multidisciplinary research teams, providing resources to new researchers, and necessary guidance to other members involved in the research project. As an educator, nurse scientist bridges the gap between research discovery and implementation, and is responsible for conducting and disseminating joint research (15). Mentoring new researchers to become part of the research team, directing training and supervising education, and organizing and planning interdisciplinary meetings and events are all manifestations of being an educator (Liu et al., 2020) (31). In addition, CRNs need to ensure that subjects are well informed about the goals and procedures of the trial (31), providing subject education and counseling. CRNs also need to alleviate the fear and hesitation of subjects due to their lack of knowledge about clinical trials.

### 3.4 Competencies for CRNs

The competencies corresponding to the characteristics of caregiver, protector, and coordinator of subjects have been more explored in studies, and the competencies required for the rest have been less mentioned in studies. As caregivers and protectors of subjects, CRNs need to possess clinical trials knowledge, skills, and literacy (Li et al., 2020), including basic knowledge of pharmacology and clinical expertise. At the same time, clinical thinking and analytical skills are also required for CRNs. Keen clinical judgment and professional leadership are also valued in continuous practice (21). As a coordinator, Deng et al. (2023) found that communication and coordination skills were the necessary professional literacies, including communication and coordination, and teamwork of subjects. On the other hand, Li et al. (2020) found that CRNs should pay more attention to the advanced level of competency development, such as the interpretation of clinical trial results, the reading of literature, and other scientific research skills. In a cross-sectional study (24), a CRN development framework was established in collaboration with clinical trial training consultants, enabling CRNs to be knowledgeable about regulatory requirements, the procedures of establishing trust with subjects, as well as guidance on drug development, experimental design, intellectual property, and recruitment procedures.

## 4 Discussion

This scoping review explored the characteristics of CRNs identified in 26 articles. The characteristics of CRNs were first described, and then the abilities corresponding to the roles of CRNs are discussed.

Regarding the first research question, the results of this review indicate that CRNs assume the roles of participants, managers, caregivers, protectors, coordinators and educators. CRNs have evolved from mere data collectors to integrated members of clinical trial teams, contributing to the generation of clinical evidence in addition to their nursing roles in clinical practice, maintaining subject safety, study coordination and management. Characteristics of CRNs are complex, among which the findings on the most important role vary from study to study. Several studies have shown that clinical trials management is the primary role (13, 29), whereas Kao (26) found that subject protection was the most recognized. Different studies did not have the same findings on subject protection. In terms of obtaining informed consent from subjects, different studies have given qualifications on the prerequisites for informed consent. Emanuel (36) argued that informed consent is an autonomous decision to participate in a clinical trial or not, rather than being coerced. In New Zealand (29), informed consent can only be obtained when subject is conscious, and for unconscious subjects, CRNs are usually required to communicate with family members to decide. This scoping review has newly discovered the cultural protection of subjects in research (9), and this finding can guide the future emphasis on cultural differences in clinical nursing research, minimize the negative cultural experience of subjects in the process of clinical trials, and give subjects basic cultural respect and humanistic care. Ethnic minority subjects from different cultural backgrounds have differences in language communication, cultural customs, social functions and roles. For different countries, the differences in common languages, the impact of national culture and the social and economic barriers make CRNs in different countries need to discard prejudices according to their own cultural background, and protect subjects with tolerance and respect in clinical trials.

Regarding the second question, the results indicate three competencies: (1) clinical trials knowledge, skills and literacy; (2) communication and coordination; (3) advanced research skills. As a caregiver and protector of subjects, the competencies are mostly focused on nursing practice. As a coordinator, well-honed communication and coordination skills help CRNs appropriately handle the communication between multidisciplinary teams. As a contact for subjects interested in or participating in a clinical trial study, expertise in nursing and understanding of the healthcare system allow CRNs to quickly establish trust and rapport with subjects, bridge the information gap between researchers and subjects, and effectively relieve their concerns and ensure their safety and comfort throughout the trial process based on expectations. The development of advanced research skills is more conducive to CRNs’ understanding of clinical trials and the accuracy of results interpretation (23, 37), which is more conducive to the success of research. Whereas advanced research competencies are beneficial to the scientific nature of nursing research, nurses with strong research competencies can contribute to evidence-based practice, improve subject outcomes and help advance medical outcomes. By cultivating research competencies, CRNs can take on important roles on research teams, influence and lead nursing-related decisions on the research team, integrate clinical trial with nursing practice, improve the quality of clinical care, and contribute to the advancement of nursing.

## 5 Conclusion

The results of this scoping review reflect the characteristics and competencies requirements of CRNs, which form a comprehensive overview of the role of the CRNs. Understanding the characteristics of CRNs is critical to healthcare organizations, researchers’ contributions to optimizing clinical practice, and improving treatment outcomes for subjects. Further research is still needed in the future to explore the gaps between the roles of CRNs and the importance of each role, and to create a clear occupational scope and duties to improve the quality of clinical trials and promote clinical evidence-based practice.

## 6 Limitations

Although five commonly used databases were applied for the literature search, studies on characteristics of CRNs in other databases may have been excluded. Additionally, the title and abstract review may be insufficient to reflect the initial findings of all studies effectively, and some relevant articles may have been removed. In this scoping review, the CRNs’ role was described, however the level of importance among the revealed characteristics in the result, was not clarified. Finally, only Chinese and English literature was selected in this review process, which may lead to incomplete literature retrieval.

## Data availability statement

The raw data supporting the conclusions of this article will be made available by the authors, without undue reservation.

## Author contributions

YX: Conceptualization, Data curation, Formal analysis, Writing – original draft. XW: Conceptualization, Formal analysis, Writing – review & editing. CZ: Conceptualization, Formal analysis, Writing – review & editing. WY: Conceptualization, Project administration, Supervision, Writing – review & editing. XC: Conceptualization, Methodology, Project administration, Supervision, Writing – review & editing. WL: Conceptualization, Methodology, Project administration, Supervision, Writing – review & editing.

## Footnotes

^1^https://www.cnki.net/

^2^http://www.wanfangdata.com.cn/index.html
