# Supplementary material for: Characteristics and duties of clinical research nurses: a scoping review
Source: Front Med (Lausanne). 2024 Jan 18;11:1333230. doi: 10.3389/fmed.2024.1333230 (PMC10830629; doi:10.3389/fmed.2024.1333230)
Supplement: Supplementary file 1 [file Data_Sheet_1.docx]

Supplementary Material

# Supplementary Figures and Tables

## Supplementary Figures


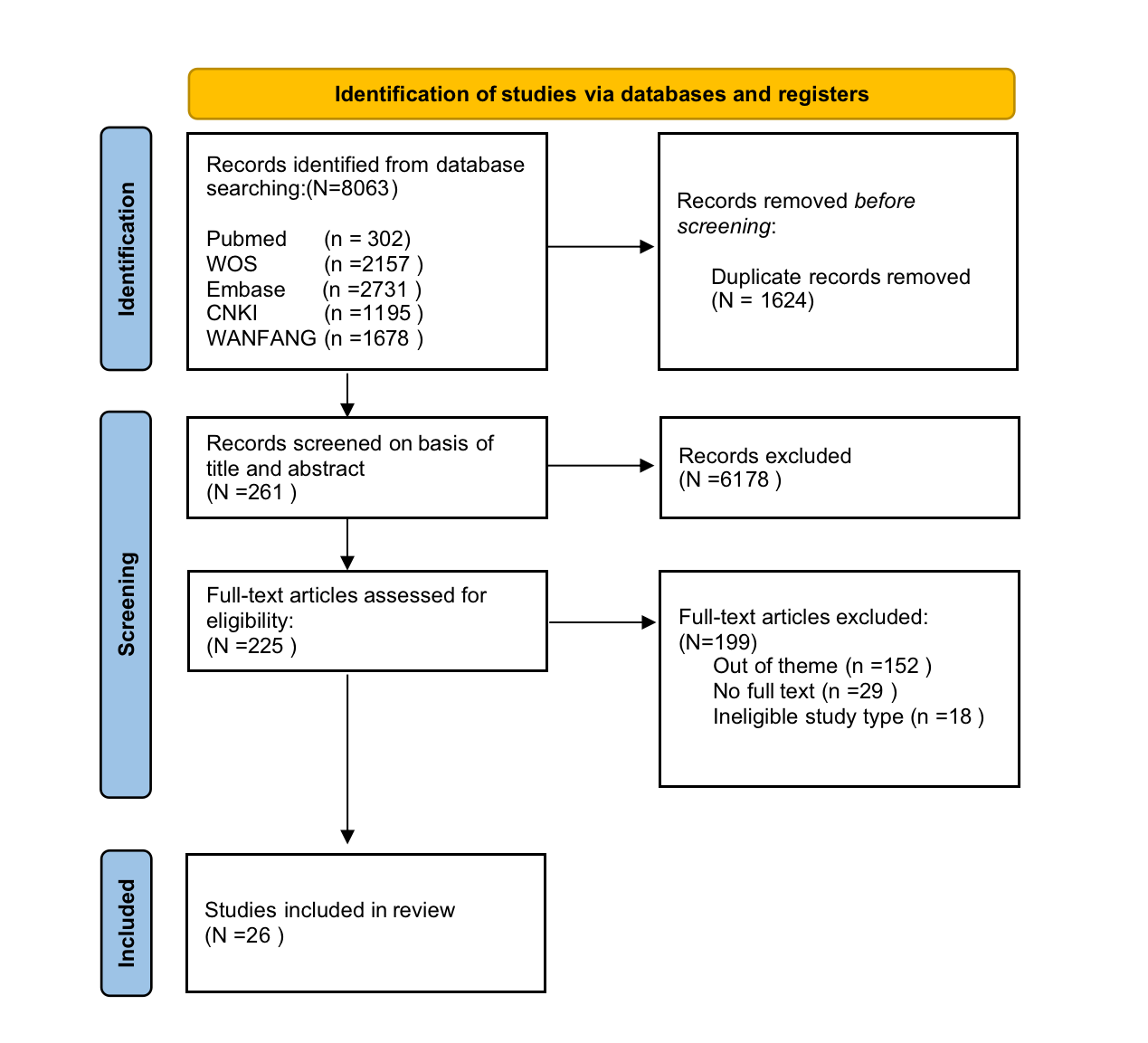


**Supplementary Figure 1.** Flowchart of the literature screening process.

## Supplementary Tables

**Supplementary Table 1.** General characteristics of the included literature

| Authors, publication year, country | Type of study | Research theme | Main findings |
| --- | --- | --- | --- |
| (Barthow et al., 2015)  New Zealand | Qualitative study | Role of the research nurse | Explaining the purpose of the study, obtaining participant consent, using clinical assessment tools with research instruments, using information technology for data management and analysis. |
| (Beer et al., 2022)  Australia | Qualitative study | Role of the research nurse | Acting as an enabler to address systemic barriers; acting as a facilitator to address practitioner barriers; acting as a navigator to address subject barriers. |
| (Bevans et al., 2011)  United States | Quantitative research | Role of the research nurse | Providing direct clinical and research care to individual participants; clinical practice; facilitating communication with the research team, communicating the impact of study procedures on subjects, coordinating activities to minimize risk to participants. |
| (Brant et al., 2015)  United States | Qualitative research | Role of the research nurse | The mentor is the primary role; other roles include adjunct faculty at the local school of nursing, cancer committee members, and the principal investigator. |
| (Carter et al., 2020)  United States | Qualitative research | Role of the research nurse | Conducting and disseminating joint research, translating evidence into practice, developing formal education programs for health system nurses, scholarly activity between health systems, and increasing the visibility and valuation of postdoctoral nurses. |
| (Cassidy et al., 1991)  United States | Qualitative research | Role of the research nurse | Clinical trials, administrative tasks, adherence to protocol guidelines, conducting subject assessments, encouraging subject trust and reassurance, providing messaging, establishing ongoing open lines of communication, completing treatment protocols, toxicity observation and reporting, follow-up tracking, proper documentation of study records. |
| (Catania et al., 2012)  Italy | Quantitative study | Role of the research nurse | Much involvement in actual nursing care with little attention to data management and organizational activities. Involved in experimental drug administration, protocol implementation, partially involved in informed consent process. |
| (Dube et al., 2017)  United States | Qualitative research | Role of the research nurse | Assessment and planning of care related to clinical research before, during and after surgery. |
| (Ecklund et al., 1999)  United States | Qualitative research | Role of the research nurse | Protocol development and implementation, data management, subject tracking, communication, personnel management, organization and planning of staff meetings, fiscal management, and community outreach. |
| (Gibbs et al., 2012)  United Kingdom | Qualitative research | Role of the research nurse | Coordinating the day-to-day management of the trial and ensuring that the study is conducted in accordance with relevant legislation, research protocols and guidelines. The most recognized skills and responsibilities include screening, recruiting and obtaining informed consent from subjects and/or relatives. |
| (Green et al., 2011)  United Kingdom | Qualitative research | Role of the research nurse | Acting as a direct caregiver; being a key contact for trial participants; organizing research ethics committees and research and development submissions, and all related correspondences. |
| (Hølge-Hazelton et al., 2016)  United Kingdom | Qualitative research | Role of the research nurse | Use of knowledge in practice, clinical thinking and analytical skills, clinical judgment and decision-making skills, professional leadership and clinical investigation, coaching and mentoring skills, research skills and changing practice. |
| (Johnson et al., 2010)  United Kingdom | Qualitative research | Role of the research nurse | Effective communication with members of the multidisciplinary team and good relationships with all departments and trial participants involved in the trial. |
| (Kao et al., 2015)  China | Quantitative research | Role of the research nurse | The role related to "subject protection" had the highest level of agreement, followed by "study coordination and management", "clinical care of subjects", "clinical care of subjects", "clinical care of subjects", "clinical care of subjects", and "clinical care of subjects", and "advanced practice nursing functions". |
| (Lavender et al., 2019)  United Kingdom | Qualitative research | Role of the research nurse | Clinical research nurses play an expert role in recruiting subjects and caring for subjects in clinical trials, which requires specialized education and training. |
| (Ledger, 2008)  United Kingdom | Quantitative research | Competencies required of research nurses | Individual development framework: in collaboration with an external clinical research training consultant, a course specifically designed to equip research nurses with knowledge of regulatory requirements as well as trust processes and procedures, and guidance on drug development, trial design, intellectual property rights, and recruitment procedures. |
| (Legor et al.,2021)  United States | Qualitative research | Role of the research nurse | As advocates; as care coordinators; as educators. |
| (Wilkes et al., 2012)  Australia | Quantitative research | Role of the research nurse | Respondents' role domains had the highest frequency scores for the informed consent process, followed by implementation and evaluation, and data management. |
| (Liu et al., 2020)  China | Quantitative research | Role of the research nurse | Clinical practice;clinical trial management-protocol;subject management;pharmaceutical management;specimen management;equipment management;documentation management; coordination; subject protection; education. |
| (Li et al., 2020)  China | Qualitative research | Competencies required of research nurses | A system of core competency indicators for research nurses was established: basic knowledge, skills, and literacy; clinical practice; study management;coordinated management and ongoing management of studies; human subjects protection; scientific contribution. |
| (Deng et al., 2023)  China | Quantitative research | Competencies required of research nurses | A training system for research nurses was constructed, consisting of four level of indicators: "clinical trial-related professional knowledge", "nursing theoretical knowledge", "nursing professional skills", "professionalism", 14 secondary indicators, and 56 tertiary indicators. |
| (Veal et al., 2017)  United Kingdom | Quantitative research | Role of the research nurse | Collection and handling of blood samples; transportation of clinical samples; administration of new drugs or procedures; administration of investigational medicines; informed consent of participating subjects; prescribing of investigational medicines in the clinical trial setting. |
| (McCabe et al., 2019)  United States | Qualitative Research | Role of the research nurse | Comprehensive care of participants; training and education of subjects and staff; contribution to clinical science; unique combination of clinical and critical thinking skills; clinical practice. |
| (Mackle et al., 2019)  New Zealand | Qualitative research | Role of the research nurse | Managing research trials; obtaining subject consent; balancing subject needs with research needs; bridging gaps to achieve research. |
| (A Fisher et al., 2022)  United States | Quantitative research | Role of the research nurse | Monitoring study participants for potential adverse events; collecting study endpoint data; providing nursing leadership within the interdisciplinary team；participation in clinical, unit, and/or protocol rounds. |
| (Backman Lönn et al., 2022）  Sweden | Quantitative research | Role of the research nurse | CRNs are involved in pre-study activities, study implementation and evaluation to various degrees. Concerned informed consent and managing the investigational products were rated as most important. |

**Supplementary Table 2.** Characters of the CRNs

| Role | Description | Citation |
| --- | --- | --- |
| Participants and administrators of clinical trials | 1.Participation in clinical trials: Management of clinical and research execution activitie | (Barthow et al., 2015；Bevans et al., 2011；Cassidy et al., 1991) |
|  | 2.Follow uniform clinical guidelines | (Bevans et al., 2011；Ecklund et al., 1999) |
|  | 3. Clinical trial management  1)Data management  2)Specimen management  3)File management  4)Drug management  5)Facility management  6)Subject management | (Dube et al., 2017；Barthow et al., 2015；Bevans et al., 2011；Catania et al., 2012；Ecklund et al., 1999；Gibbs et al., 2012；Green et al., 2011；Liu et al., 2020；Veal et al., 2017;A Fisher et al., 2022;Backman Lönn et al., 2022) |
| Caregivers and protectors of subjects | 1.Direct care for subjects | (Dube et al., 2017；Bevans et al., 2011;Cassidy et al., 1991;Brant et al., 2015;McCabe et al., 2019;Lavender et al., 2019) |
|  | 2.Subject protection | (Legor et al.,2021;Barthow et al., 2015;Cassidy et al., 1991;Gibbs et al., 2012；Kao et al., 2015；Liu et al., 2020；Mackle et al., 2019;Wilkes et al., 2012;A Fisher et al., 2022;Backman Lönn et al., 2022) |
| Coordinator of research teams | Facilitates interdisciplinary team communication and collaboration | (Cassidy et al., 1991;Liu et al., 2020；Mackle et al., 2019;Beer et al., 2022;A Fisher et al., 2022  ) |
| Educator | Guide training and education | (Legor et al.,2021;Catania et al., 2012；Liu et al., 2020) |

# Supplementary material :Database searching

*Appendix I. Database searching*

| **Database** | **Search Terms** |
| --- | --- |
| **WOS** | ((((((TS=("research nurse coordinator*" )) OR TS=("research nurse*" )) OR TS=("nurse researcher*")) OR TS=("nurse scientist*")) OR TS=("nursing scientist*")) OR TS=("clinical trial nurse*")) OR TS=("clinical research nurse*") AND DT=(Article) |
| **Embase** | ('research nurse coordinator*':ab,ti OR 'research nurse*':ab,ti OR 'nurse researcher*':ab,ti OR 'nurse scientist*':ab,ti OR 'nursing scientist*':ab,ti OR 'clinical trial nurse*':ab,ti OR 'clinical research nurse*':ab,ti) AND article:it AND ([embase]/lim OR [medline]/lim) |
| **PUBMED** | ("research nurse coordinator*"[Title/Abstract] OR "research nurse*"[Title/Abstract] OR "nurse researcher*"[Title/Abstract] OR "nurse scientist*"[Title/Abstract] OR "nursing scientist*"[Title/Abstract] OR "clinical trial nurse*"[Title/Abstract] OR "clinical research nurse*"[Title/Abstract]) AND (books docs [Filter] OR clinical trial[Filter] OR randomized controlled trial[Filter]) |
